# Assessment of 10-Year Left-Ventricular-Remodeling by CMR in Patients Following Aortic Valve Replacement

**DOI:** 10.3389/fcvm.2021.645693

**Published:** 2021-03-22

**Authors:** Nina Rank, Lukas Stoiber, Mithal Nasser, Radu Tanacli, Christian Stehning, Jan Knierim, Felix Schoenrath, Burkert Pieske, Volkmar Falk, Titus Kuehne, Alexander Meyer, Sebastian Kelle

**Affiliations:** ^1^Department of Internal Medicine & Cardiology, German Heart Center Berlin, Berlin, Germany; ^2^Department of Cardiothoracic & Vascular Surgery, German Heart Center Berlin, Berlin, Germany; ^3^Philips Healthcare, Hamburg, Germany; ^4^DZHK (German Centre for Cardiovascular Research), Partner Site Berlin, Berlin, Germany; ^5^Department of Internal Medicine and Cardiology, Charité-Universitätsmedizin Berlin, Berlin, Germany; ^6^Department of Cardiovascular Surgery, Charité-Universitätsmedizin Berlin, Berlin, Germany; ^7^Department of Health Science and Technology, Eidgenössische Technische Hochschule Zurich, Zurich, Switzerland; ^8^Institute for Cardiovascular Imaging Science and Computational Modelling, Charité-Universitätsmedizin Berlin, Berlin, Germany

**Keywords:** cardiac magnetic resonance imaging, ventricular remodeling, aortic valve disease, aortic valve replacement, aortic stenosis, aortic regurgitation

## Abstract

**Aims:** Aortic valve replacement (AVR) may result in reverse cardiac remodeling. We aimed to assess long-term changes in the myocardium following AVR by Cardiac Magnetic Resonance Imaging (CMR).

**Methods:** We prospectively observed the long-term left ventricular (LV) function and structure of 27 patients with AVR [*n* = 19 with aortic stenosis (AS); *n* = 8 with aortic regurgitation (AR)] by CMR. Patients underwent CMR before, as well as 1, 5, and 10 years after AVR. We evaluated clinical parameters, LV volumes, mass, geometry, ejection fraction (EF), global myocardial longitudinal strain (MyoGLS), global myocardial circular strain (MyoGCS), hemodynamic forces (HemForces), and Late Gadolinium Enhancement (LGE).

**Results:** The median of LVMI, EDVI, and ESVI decreased in both groups. Patients with AR had higher initial values of EDVI and ESVI and showed a more prominent initial reduction. In AS, MyoGLS improved already after 1 year and remained constant afterward, whereas, in AR no improvement of MyoGLS was found. MyoGCS remained unchanged in the AS group but deteriorated in the AR group over 10 years. Ejection fraction (EF) was higher in AS patients compared to AR 10 years post-AVR. Late gadolinium enhancement (LGE) could be found more frequently in AS patients.

**Conclusion:** CMR was well suited to investigate myocardial changes over a 10-year follow up period in patients with aortic valve disease. Regarding the long-term functional changes following AVR, patients with AR seemed to benefit less from AVR compared to AS patients. Fibrosis was more common in AS, but this did not reflect functional evolution in these patients. Close monitoring seems indispensable to avoid irreversible structural damage of the heart and to perform AVR at an appropriate stage.

## Introduction

Long-term pressure and volume overload in aortic stenosis (AS) and aortic regurgitation (AR), respectively induce either concentric or eccentric remodeling of the left ventricle (LV) (1–4).

The resulting interstitial fibrosis and impaired filling cause progressive LV dysfunction and increased mortality in patients with aortic valve disease (AVD) (3, 5, 6). Multiple studies have shown a reverse effect on cardiac remodeling after aortic valve replacement (AVR). However, less is known about the effect of AVR on long-term LV reverse remodeling (3, 7–9). Previous studies demonstrated a link of myocardial fibrosis and impaired outcome after AVR, most likely due to the incomplete reversal of fibrotic deposition despite an improvement in cardiac function (10). While fibrosis is an important element of adverse LV remodeling, it is now thought that an inflammatory response as well as vascular factors play a crucial role in the long-term maladaptive remodeling process (11).

Cardiovascular magnetic resonance imaging (CMR) provides crucial information on various elements within the ventricle and can assess the plasticity response following AVR (12). Contrast enhanced MRI techniques as Late Gadolinium Enhancement (LGE) which visualize fibrotic extent are used to predict adverse events in patients with AVD (13). Moreover, CMR-derived myocardial deformation imaging has rapidly improved over the last decade and now reflects the global and regional changes of the myocardium with higher sensitivity than conventional imaging techniques (14). The predictive power of deformation imaging in the context of AVR remains however unclear. Available long-term follow-up (FU) studies focusing on LV-remodeling have usually been based on echocardiography (15, 16). However, CMR offers higher spatial resolution and higher inter-study reproducibility and has thus become the gold standard for LV-volume quantification (17–20). Feature-tracking techniques enable the assessment of myocardial deformation imaging as strain. Myocardial strain is a more robust tool to detect systolic dysfunction than ejection fraction (EF), in particular in the presence of LV-hypertrophy (21, 22). Use of the sphericity index (SI) has been an effort to simply assess LV shape and function. An increased SI is associated with eccentric remodeling following myocardial injury, volume or pressure overload and a worse outcome after AVR (23, 24). LV-Hemodynamic forces and Intraventricular Pressure Gradients (IVPG) are modern techniques to detect clinically inapparent myocardial dysfunction. Our group previously described LV hemodynamic forces as a non-invasive technique that might detect myocardial dysfunction at an early stage, when traditional parameters such as volume and ejection fraction are still unchanged (25). The aim of our study was to monitor the LV-remodeling after AVR in patients with severe AS or AR over 10 years. An understanding of the immediate and long-term course might change the perspective of optimal timing of AVR and opens the possibility for future studies in the field.

## Methods

### Study Population

This trial was planned as a prospective, observational study. A total of twenty-seven (*n* = 27) patients with the clinical indication for aortic valve replacement due to either severe AS or AR underwent aortic surgery between 2002 and 2006. The study was approved by the local institutional review board (Charité-Universitätsmedizin Berlin: EA2/077/14) in accordance with all the ethical standards and written informed consent was provided by all patients before inclusion. All patients underwent CMR examinations, echocardiographic and clinical assessment before (*t* = 0), as well as 1, 5, and 10 years after surgery.

### Clinical Assessment

Patients were questioned about their subjective physical capacity (SPC) on a scale of 1 (very good) to 5 (very bad) and their New York Heart Association (NYHA) class was assessed (26).

### CMR

At all four points in time CMR scans were available for all patients. Data were acquired either on 1.5 Tesla or 3 Tesla clinical MR systems (1.5 T: Gyroscan NT, Achieva; 3 T: Intera, Ingenia; all Philips Healthcare, Best, the Netherlands). The CMR protocol included standard balanced Fast Field Echo (bFFE) cine CMR with at least three short-axis slices, and one slice in 2-chamber, 3-chamber, and 4-chamber orientation, respectively, as well as LGE (27). Typical imaging parameters were as follows: acquired voxel size (AVS) 1.80 × 1.70 × 8 mm^3^, reconstructed voxel size (RVS) 1.50 × 1.50 × 8 mm^3^, FOV 380 × 350 mm^2^, echo time (TE) 1.5 ms (1.5 and 3 T), time to repetition (TR) = 3.0 ms (1.5 and 3 T), flip angle 60°, bandwidth = 962 Hz/pixel, parallel acquisition technique = SENSE factor 2.0. Similar imaging parameters were employed at 3 T, for a flip angle = 45°, and a bandwidth of 1,803 Hz/pixel. A dual transmit Radio Frequency coil with volume adaptive B1 shimming was employed on the 3 T Ingenia system.

### Echocardiography

Additionally, transthoracic echocardiographic studies were performed at all time-points. Standard views and Doppler flow-based measurements where obtained to quantify mean pressure gradient ΔPm over the aortic valve.

### Image Analysis

All CMR images were analyzed by the same experienced reader. Epi- and endocardial contours were marked manually at end-systole and end-diastole in all orientations. Volume and functional parameters [EDV, ESV, EF, Global myocardial longitudinal (MyoGLS) and circumferential strain (MyoGCS), hemodynamic forces (HF)] were derived from cine images using QStrain software from Medis (Version 2.1, Medis, Leiden, The Netherlands). Myocardial mass was calculated using the American Society of Echocardiography (ASE)-recommended area-length formula described by Schiller et al. (28). LV-Mass, EDV and ESV were indexed to body surface area (BSA), further denoted as LVMI, EDVI, and ESVI.

The sphericity index (SI) was calculated as follows (29):

$$\begin{array}{l} SI\;=\;\frac{EDV}{\frac{4\pi }{3}\left(\frac{4Ch\;length}{2}\right)3} \end{array}$$

whereas, the four-chamber (4Ch) length was assessed manually at end-diastole as the distance from the apex to the middle of the mitral annulus in the 4Ch view.

### Statistical Analysis

Statistical analyses were performed using R (version 3.3.3). Continuous variables were tested for normal distribution with the Shapiro-Wilk test. For our baseline analysis the R package “compareGroups” (version 3.2.4) was used (30). Normally distributed variables were reported as mean ± standard deviation and compared by the student's *t*-test. Non-normally distributed variables were presented as median and interquartile range and compared by the Kruskal-Wallis test. Categorical values were expressed as a number of patients and percentages and compared by the Chi-squared or exact Fisher test when necessary.

Not all our variables were normally distributed at each point in time. Additionally, the development of some ordinal scaled variables (NYHA, SPC) was investigated. Therefore, the median and interquartile ranges (IQR) were considered the most informative statistics regarding the assessment of the development of these variables over time. For statistical analysis the ordinal categories of NYHA and SPC were assigned rank values (for NYHA: ranks 1–4 for classes I-IV, for SPC: ranks 1–5 for categories 1–5, respectively).

The parameters were further compared between the AS and the AR group at each point in time with the Wilcoxon rank-sum test (31). Additionally, for both groups separately, the parameters were compared between the single points in time with ANOVA-Type statistic from the R package “nparLD” (version 2.1), a package designed for non-parametric analysis of longitudinal data (32). To control for Type I error, *p*-values were adjusted with Bonferroni correction (33).

## Results

### Study Population

Between 2002 and 2006, 100 patients underwent AVR. Over the 10-year FU period, thirty-four (*n* = 34) of those had to be excluded of our study group. The main reasons for dropout are illustrated in Supplementary Figure 1.

Of the remaining sixty-six (*n* = 66) patients, twenty-seven (*n* = 27) had an MRI at 4 time points and were thus included in the present analysis. This group comprises nineteen (*n* = 19) patients with AS and eight (*n* = 8) patients with AR. All patients were male. Patients with combined aortic valve disease were assigned to the group which corresponded to their leading valve pathology: Six patients were assigned to AS- and two patients to the AR-group.

A detailed analysis on the prosthetic valve types used as wells as on the indications for surgery can be found in the Supplementary Material of our manuscript. Apart from the age, no significant differences in the baseline characteristics between the groups could be observed. The mean age was 65.1 ± 6.6 years in the AS group and 56.7 ± 6.6 years in the AR group (*p* = 0.01). Baseline characteristics are listed in Table 1.

**Table 1:** Baseline characteristics of the study population.

**Parameter**	**All patients** ***n* = 27**	**AR** ***n* = 8**	**AS** ***n* = 19**	***p*-value**
Male, no. (%)	27 (100)	8 (100)	19 (100)	–
Age, mean ± std	62.6 ± 7.6	56.7 ± 6.6	65.1 ± 6.6	0.010
BMI, mean ± std	27.5 ± 3.5	26.7 ± 2.2	27.8 ± 3.9	0.377
NYHA class, no. (%)	–	–	–	1.000
1	11 (40.7)	3 (37.5)	8 (42.1)	1.000
2	9 (33.3)	3 (37.5)	6 (31.6)	1.000
3	7 (25.9)	2 (25.0)	5 (26.3)	1.000
Beta-blocker, no. (%)	15 (55.6)	3 (37.5)	12 (63.2)	0.398
ACEI, no (%)	11 (40.7)	5 (62.5)	6 (31.6)	0.206
ARB, no. (%)	3 (11.1)	2 (25.0)	1 (5.26)	0.201
Calcium channel blockers, no. (%)	3 (11.1)	2 (25.0)	1 (5.26)	0.201
Diuretics, no. (%)	10 (37.0)	3 (37.5)	7 (36.8)	1.000
HMG-CoA-I no. (%)	8 (29.6)	2 (25.0)	6 (31.6)	1.000
Hypertension, no. (%)	16 (59.3)	6 (75.0)	10 (52.6)	0.405
Dyslipidemia, no. (%)	24 (88.9)	6 (75.0)	18 (94.7)	0.201
Diabetes mellitus, no. (%)	2 (7.41)	0 (0.00)	2 (10.5)	1.000
Arteriosclerotic heart disease, no. (%)	2 (7.41)	0 (0.00)	2 (10.5)	1.000

*ACEI, angiotensin-converting-enzyme inhibitors; AR, aortic regurgitation; ARB, angiotensin-II receptor blockers; AS, aortic stenosis; BMI, body mass index; HMG-CoA-I, HMG-CoA reductase inhibitors; NYHA, New York Heart Association Functional Classification*.

### Clinical Parameters

**Figure 1:**
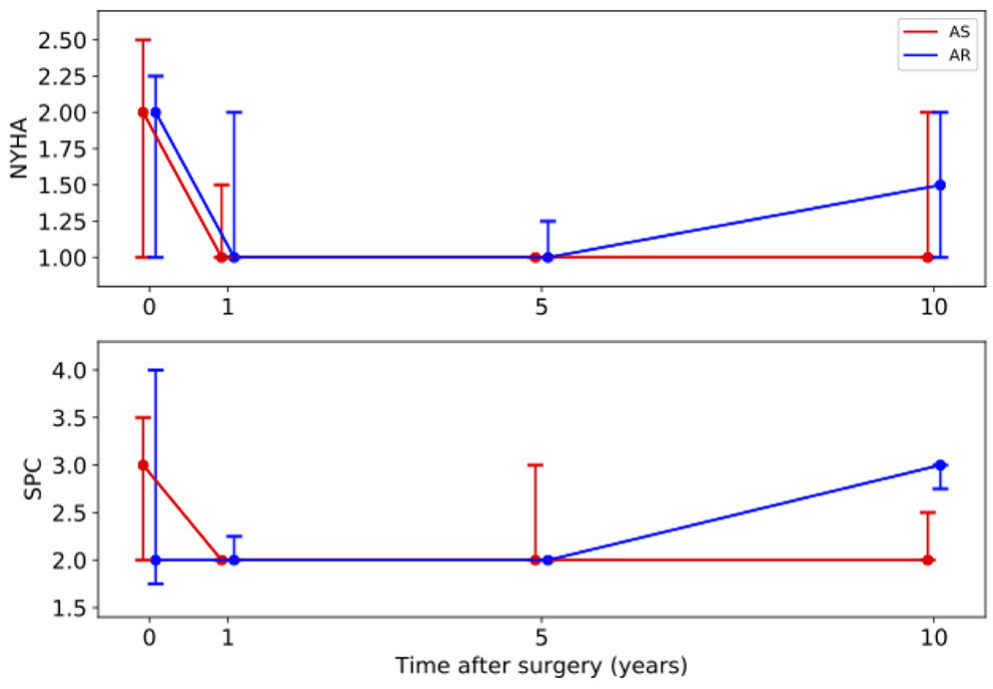
Long-term development of the medians with interquartile ranges of clinical parameters. Red line – patients with aortic stenosis (AS). Blue line – patients with aortic regurgitation (AR). NYHA, New York Heart Association Functional Classification; SPC, subjective physical capacity.

The development of the clinical parameters is shown in Figure 1. For both groups the median of the NYHA class was 2 (AS: 1–2.5; AR: 1–2.3) before surgery. In the AS group it significantly improved to 1 (1–1) within the following 5 years (*p* = 0.007) and stayed stable over 10 years. In the AR group it improved non-significantly to 1 (1–2) but deteriorated to 1.5 (1–2) at year ten. In the AS group the SPC significantly improved from 3 (2–3.5) at baseline to 2 (2–2) within the first year (*p* = 0.020) and stayed stable afterwards. In the AR group it first stayed at 2 (1.8–4) but deteriorated significantly from 2 (2–2) at year 5 to 3 (2.75–3) at year 10 (*p* = 0.040).

### Anatomical Parameters

**Figure 2:**
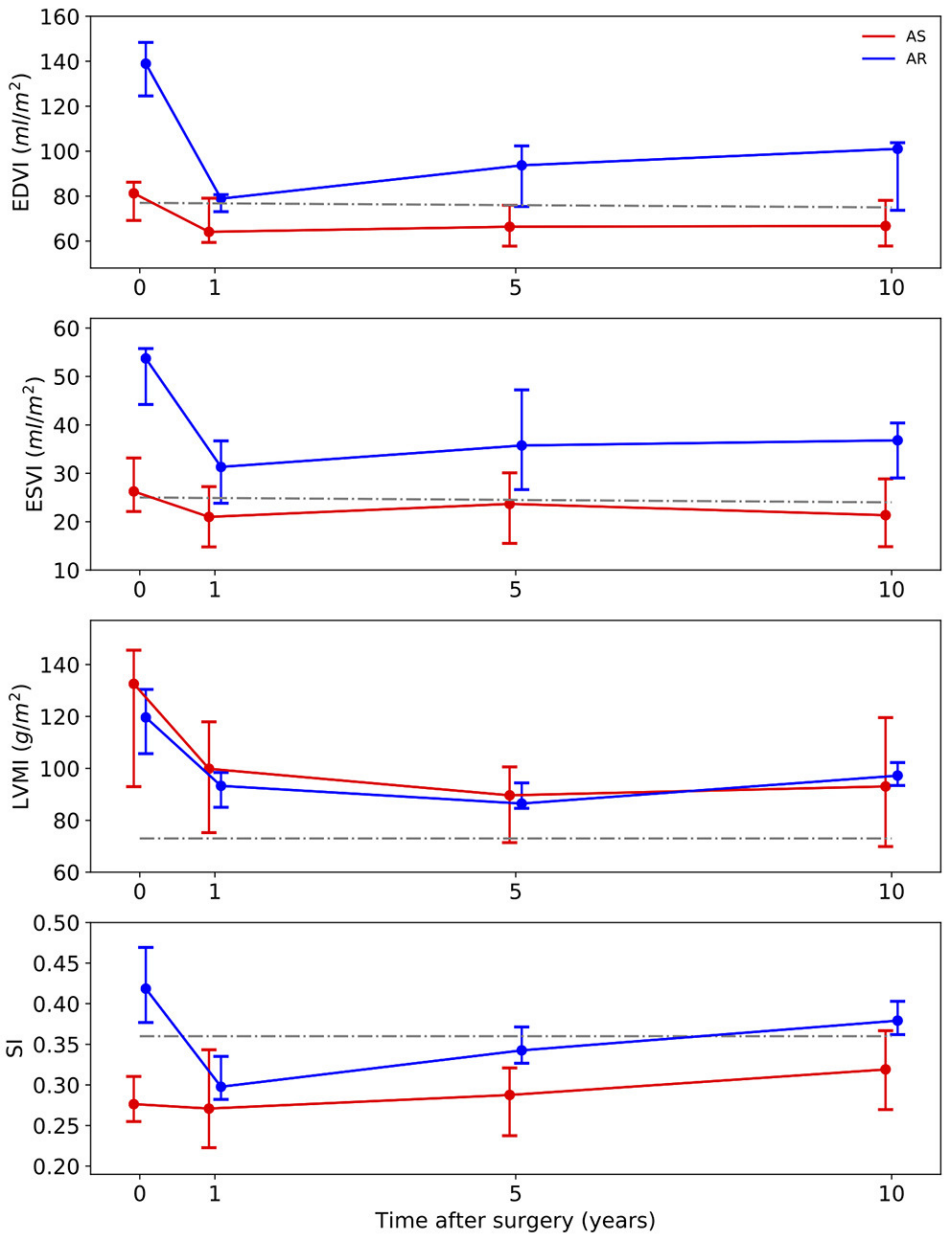
Long-term development of the medians with interquartile ranges of anatomic parameters. Red line – patients with aortic stenosis (AS). Blue line – patients with aortic regurgitation (AR). Gray line – normal values (34). EDVI, end-diastolic volume index; ESVI, end-systolic volume index; LVMI, left ventricular mass index; SI, sphericity index.

Figure 2 illustrates the results of the investigated anatomical parameters. In the AS group, EDVI only decreased slightly from 81 (69–86) ml/m^2^ at baseline to 64 (59–76) ml/m^2^ at year 1 and stayed stable afterwards. A similar behavior could be found for the ESVI. In contrast, in the AR group, the values were significantly higher at baseline [EDVI: 139 (125–148) ml/m^2^, ESVI: 54 (44–56) ml/m^2^] than in the AS group (*p* < 0.001). Both values significantly dropped 1 year after surgery [EDVI: 79 (73–81) ml/m^2^, ESVI: 31 (24–37) ml/m^2^, *p* < 0.001], but increased, though not significantly, again over the 10 post-operative years [EDVI: 101 (74–104) ml/m^2^, ESVI: 37 (29–40) ml/m^2^ at year 10].

Regarding the LVMI the AS group and the AR group showed a similar development. The median values were high above the normal values at baseline [AS: 133 (93–146) g/m^2^, AR: 120 (106–130) g/m^2^]. These values significantly decreased (*p* < 0.001) over the following 5 years [AS: 90 (71–101) g/m^2^, AR: 86 (85–94) g/m^2^ at 5-year FU], but then showed a slight increase again at year 10.

No prominent temporal changes of the SI could be observed in the AS group [0.28 (0.25–0.31) at baseline] 1 year after the surgery. A significant increase between year 1 and year 10 was noted (*p* = 0.007) though. In patients with AR, the SI was significantly higher at baseline than in the AS group (*p* = 0.018). It significantly decreased from 0.42 (0.38–0.47) before surgery to 0.30 (0.28–0.34) at year 1 (*p* = 0.013). It also showed an increasing trend to 0.38 (0.36–0.4) at year 10.

### Functional Parameters

**Figure 3:**
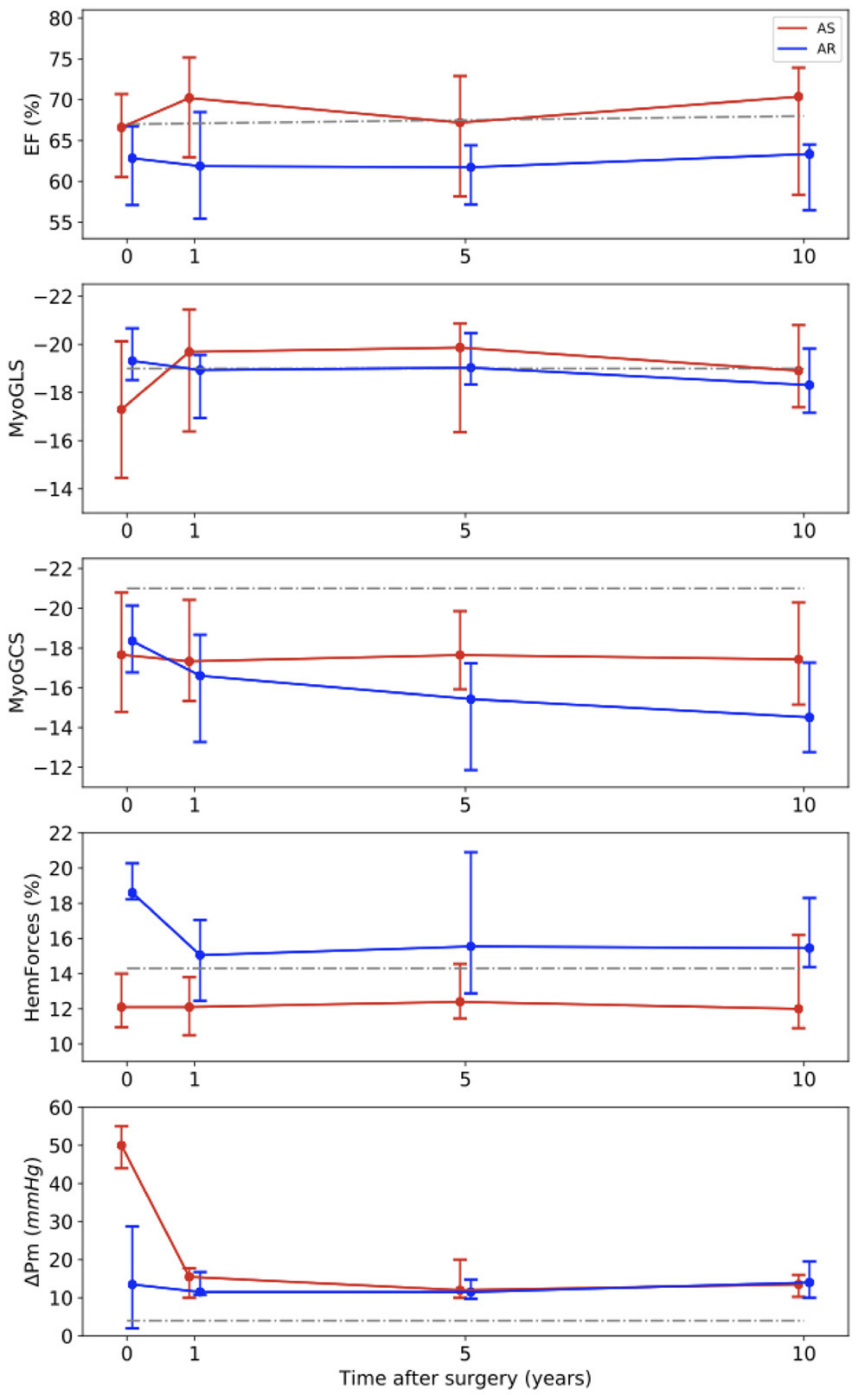
Long-term development of the medians with interquartile ranges of functional parameters. Red line – patients with aortic stenosis (AS). Blue line – patients with aortic regurgitation (AR). Gray line – normal values (34) for EF, (35) for MyoGLS and MyoGCS, (25) for HemForces, (36) for ΔPm. EF, ejection fraction; MyoGLS, myocardial global longitudinal strain; MyoGCS, myocardial global circumferential strain; HemForces, hemodynamic forces; ΔPm, mean pressure gradient across the aortic valve.

The development of the functional parameters is illustrated in Figure 3. Regarding the EF, no major changes were found after AVR, neither in the AS nor in the AR group. In the AS group, a slight increase of the EF from 66 (61–71)% at baseline to 70 (63–75)% was observed.

The MyoGLS improved from −17.3 (−20.1 to −14.5)% at baseline to −19.7 (−21.5 to −16.4)% at year 1 in the AS group. The AR group had a lower, nearly normal, MyoGLS of −19.3 (−20.7 to −18.5)% before surgery that remained stable over time. In the AS group, the median MyoGCS was −17.7 (−20.8 to −14.8)% at baseline and remained in this range for the whole observation period. In contrast, in the AR group a continuous deterioration from −18.4 (−20.1 to −16.9)% at baseline to −14.5 (−17.3 to −12.8)% at year 10 was observed. The median HemForces in the AS group were 12.1 (11.0–17.8)% at baseline and was stable over time. In the AR group, it was significantly higher at baseline than in the AS group (*p* < 0.001). The value decreased from 18.6 (18.2–20.3)% before surgery to 15.0 (12.5–17.1)% after 1 year and remained stable afterwards. The changes in functional parameters showed only trends but were not significant. In six patients, baseline echocardiographic data were incomplete. In the AS group, the median of the mean pressure gradient across the aortic valve was 50.0 (44.0–55.0) mmHg at baseline and significantly decreased to 15.5 (10.0–17.8) mmHg after 1 year (*p* < 0.001), remaining stable afterwards. In the AR group, it was largely stable over all four investigated points in time [baseline: 13.5 (2.0–28.5) mmHg]. Tables 2, 3 display medians and IQRs of all investigated parameters at all four points in time. Supplementary Table 1 shows the comparison between groups at all points in time for each variable, respectively. In Supplementary Tables 2, 3 individual points in time for each parameter were compared for AS and AR separately.

**Table 2:** Medians and interquartile ranges of clinical, anatomic and functional parameters for patients with aortic stenosis (AS) for all four points in time.

**Parameter**	**Group**	**FU = 0 years**	**FU = 1 year**	**FU = 5 years**	**FU = 10 years**
NYHA	AS	2 (1–2.5)	1 (1–1.5)	1 (1–1)	1 (1–2)
SPC	AS	3 (2–3.5)	2 (2–2)	2 (2–3)	2 (2–2.5)
EDVI (ml/m^2^)	AS	81.3 (69.2–86.2)	64.1 (59.4–79.1)	66.4 (57.8–75.9)	66.7 (57.8–78.1)
ESVI (ml/m^2^)	AS	26.3 (22.1–33.2)	21.0 (14.8–27.2)	23.7 (15.5–30.1)	21.4 (14.9–28.8)
LVMI (g/m^2^)	AS	132.6 (93.0–145.5)	99.9 (75.2–117.9)	89.6 (71.4–100.6)	93.0 (69.9–119.6)
SI	AS	0.28 (0.25–0.31)	0.27 (0.22–0.34)	0.29 (0.24–0.32)	0.32 (0.27–0.37)
LVEF (%)	AS	66.6 (60.5–70.7)	70.2 (63.0–75.2)	67.2 (58.2–72.9)	70.4 (58.4–73.9)
MyoGLS (%)	AS	−17.3 (−20.1 to −14.5)	−19.7 (−21.5 to −16.4)	−19.9 (−20.9 to −16.4)	−18.9 (−20.8 to −17.4)
MyoGCS (%)	AS	−17.7 (−20.8 to −14.8)	−17.3 (−20.4 to −15.3)	−17.7 (−19.9 to −15.9)	−17.4 (−20.3 to −15.2)
HemForces (%)	AS	12.1 (11.0–14.0)	12.1 (10.5–13.8)	12.4 (11.5–14.6)	12.0 (10.9–16.2)
ΔPm (mmHg)	AS	50.0 (44.0–55.0)	15.5 (10.0–17.8)	12.0 (10.0–20.0)	13.5 (10.3–16.0)

*EDVI, end-diastolic volume index; LVEF, ejection fraction; ESVI, end-systolic volume index; FU, follow up; HemForces, hemodynamic forces; MyoGCS, myocardial global circumferential strain; LVMI, left ventricular mass index; MyoGLS, myocardial global longitudinal strain; SI, sphericity index; SPC, subjective physical capacity; ΔPm, mean pressure gradient across the aortic valve*.

**Table 3:** Medians and interquartile ranges of clinical, anatomic and functional parameters for patients with aortic regurgitation (AR) for all four points in time.

**Parameter**	**Group**	**FU = 0 years**	**FU = 1 year**	**FU = 5 years**	**FU = 10 years**
NYHA	AR	2 (1–2.5)	1 (1–2)	1 (1–1.25)	1.5 (1–2)
SPC	AR	2 (1.75–4)	2 (2–2.25)	2 (2–2)	3 (2.75–3)
EDVI (ml/m^2^)	AR	139.0 (124.6–148.4)	78.9 (73.1–80.6)	93.7 (75.4–102.3)	101.0 (73.7–103.8)
ESVI (ml/m^2^)	AR	53.7 (44.2–55.8)	31.3 (23.8–36.7)	35.8 (26.6–47.2)	36.8 (29.0–40.4)
LVMI (g/m^2^)	AR	119.6 (105.7–130.4)	93.3 (85.0–98.4)	86.4 (84.6–94.4)	97.2 (93.4–102.2)
SI	AR	0.42 (0.38–0.47)	0.30 (0.28–0.34)	0.34 (0.33–0.37)	0.38 (0.36–0.40)
LVEF (%)	AR	62.8 (57.1–66.8)	61.9 (55.4–68.5)	61.7 (57.2–64.4)	63.4 (56.5–64.5)
MyoGLS (%)	AR	−19.3 (−20.7 to −18.5)	−18.9 (−19.6 to −16.9)	−19.0 (−20.5 to −18.3)	−18.3 (−19.8 to −17.2)
MyoGCS (%)	AR	−18.4 (−20.1 to −16.8)	−16.6 (−18.7 to −13.3)	−15.4 (−17.2 to −11.9)	−14.5 (−17.3 to −12.8)
HemForces (%)	AR	18.6 (18.2–20.3)	15.1 (12.5–17.1)	15.6 (12.9–20.9)	15.5 (14.4–18.3)
ΔPm (mmHg)	AR	13.5 (2.0–28.8)	11.5 (10.8–16.8)	11.5 (9.8–14.8)	14.0 (10.0–19.5)

*EDVI, end-diastolic volume index; LVEF, ejection fraction; ESVI, end-systolic volume index; FU, follow up; HemForces, hemodynamic forces; MyoGCS, myocardial global circumferential strain; LVMI, left ventricular mass index; MyoGLS, myocardial global longitudinal strain; SI, sphericity index; SPC, subjective physical capacity; ΔPm, mean pressure gradient across the aortic valve*.

### Late Gadolinium Enhancement

The findings of LGE in our cohort are displayed in Table 4. We observed differences in LGE between both groups. At baseline, 5/19 (26%) of AS patients had LGE. This number increased to 8/19 (42%) after 10-years FU. Of these eight patients, six had detectable scar following myocardial infarction. Two had small circumscribed fibrosis. In the AR group, no LGE could be detected in the first 5 years of FU. After 10 years, 2/8 (25%) had LGE. One of these two patients a scar following myocardial infarction.

**Table 4:** Presence of late gadolinium enhancement (LGE) for patients with aortic regurgitation (AR) and aortic stenosis (AS) for all four points in time.

**Parameter**	**Group**	**FU = 0 years**	**FU = 1 year**	**FU = 5 years**	**FU = 10 years**
LGE	AR	0	0	0	2 (1)
	AS	5 (5)	6 (6)	6 (6)	8 (6)

*Values in Brackets represent ischemic LGE. FU, follow up; LGE, Late Gadolinium Enhancement*.

### Conduction Disorders

The electrocardiogram (ECG) findings in our cohort are displayed in Table 5. Overall, the majority of patients stayed in stable sinus rhythm over the 10 years following AVR: 6/8 (75%) of AR patients and 18/19 (95%) AS patients. In the AR group, atrial fibrillation and junctional rhythm accounted for the two patients without sinus rhythm. In the AS group one patient presented atrial fibrillation. Two AS patients presented right bundle branch block, but no AR patient. Left bundle branch block was found in one AR and two AS patients. No higher-grade AV Blocks were observed. No pacemaker had to be implanted.

**Table 5:** Presence of ECG changes in our cohort from baseline to follow-up.

**Parameter**	**Group**	**FU = 0 years**	**FU = 1 year**	**FU = 5 years**	**FU = 10 years**
Sinus rhythm	AR	8 (100%)	8 (100%)	8 (100%)	6 (75%)
	AS	19 (100%)	18 (95%)	19 (100%)	18 (95%)
Atrial fibrillation	AR	0	0	0	1 (13%)
	AS	0	1 (5%)	0	1 (5%)
Junctional rhythm	AR	0	0	0	1 (13%)
	AS	0	0	0	0
AV block grade I	AR	0	2 (25%)	3 (38%)	3 (38%)
	AS	1 (5%)	3 (16%)	3 (16%)	7 (37%)
AV block grade II/III	AR	0	0	0	0
	AS	0	0	0	0
Left bundle branch block	AR	0	0	1 (13%)	1 (13%)
	AS	2 (11%)	2 (11%)	2 (11%)	2 (11%)
Right bundle branch block	AR	0	0	0	0
	AS	1 (5%)	2 (11%)	2 (11%)	2 (11%)
Pacemaker	AR	0	0	0	0
	AS	0	0	0	0

*AV, Atrio-ventricular; AR, Aortic Regurgitation; AS, Aortic Stenosis*.

## Discussion

The present study used CMR to address the long-term effects of AVR on the myocardium in 27 patients with either AS or AR. We hypothesized that structural and functional changes of the LV following AVR are long-lasting and that these changes can be continuously identified by CMR.

**Figure 4:**
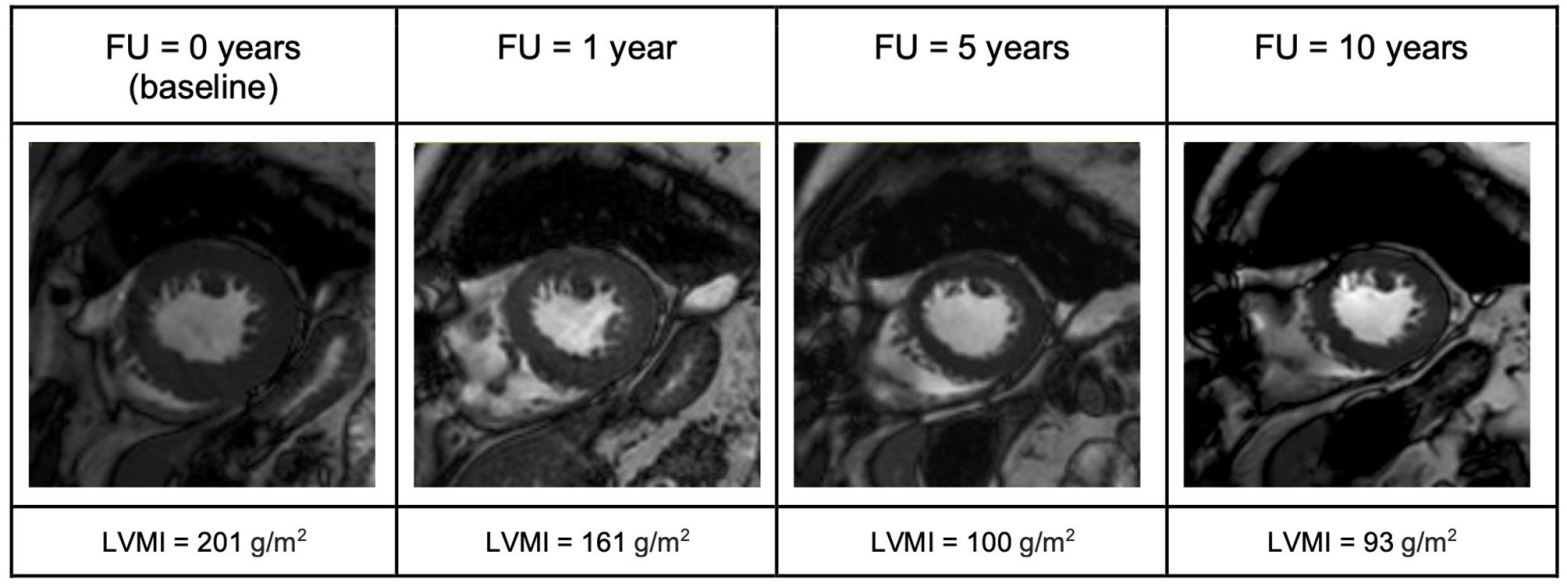
End-diastolic short-axis view illustrating evolution of LV-mass in a patient with aortic stenosis (AS) over the 10-year follow up period. FU, follow up; LVMI, left ventricular mass index.

In the AS group, AVR showed very satisfying results (Figures 1–3). Patient's symptoms improved and the subjective physical capacity increased. As expected, the mean valve gradient normalized. A substantial decrease of LVMI was also noted. The fast reduction of LV mass, as illustrated in Figure 4, is consistent with prior studies reporting a rapid decline of LV hypertrophy within the first 24 months after surgery (15). These studies also reported that hypertrophy might partially persist as it was the case in our study. Moreover, EDVI and ESVI slightly decreased and LV longitudinal strain normalized. These changes were prominent already the first year after AVR and remained stable over 10 years.

**Figure 5:**
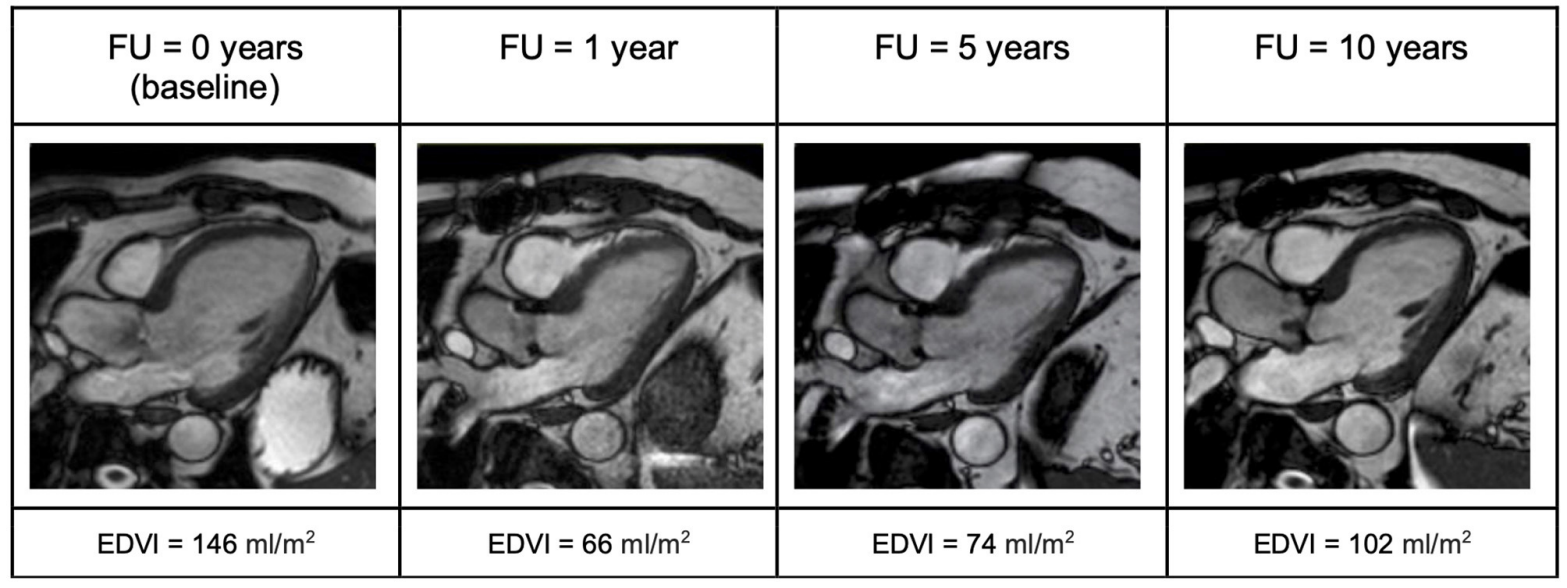
Three-chamber view illustrating evolution of LV end-diastolic volume in a patient with aortic regurgitation (AR) over the 10-year follow up period. One year after surgery a significant decrease of end-diastolic volume can be noted. However, over time this volume increases again. EDVI, End diastolic volume index; FU, follow up.

In the AR group, the long-term improvement of symptoms was less prominent. While symptoms improved within 1 year after surgery, a slight increase in NYHA class was observed 10 years after AVR. Accordingly, the subjective physical capacity declined. Before surgery, LV-EDVI and LV-ESVI were higher than in the AS group. One year after surgery, good surgical results could be seen with a substantial decrease in LV-EDVI, -ESVI, and normal LV-EF. After 5 years, increasing volumes were again observed. At 10-year FU, LV-EDVI, and –ESVI had substantially increased again (Figure 5). LV longitudinal strain was in the normal range before surgery and stayed stable in long-term FU. There was, however, no improvement of LV circumferential strain, which deteriorated over the years (Figures 1–3).

LV-Fibrosis plays an important role in the prognosis of valvular heart disease and cannot be underestimated. Several authors have provided data on LGE in AVD and its finding has priorly been linked to adverse outcome in AS patients (37). More recently, increased extracellular volume (ECV) and T1 mapping was found to be linked to worse outcome as well (38). Interestingly, 8/19 (42%) of AS patients presented LGE after 10 years. However, despite increased LGE, functional outcome was not worse in our AS patients compared to AR. In fact, LGE was even less frequent in the AR group (2/28; 25%) after 10 years, which is rather unexpected considering the beforementioned volumetric impairment. In this respect, LGE might not be the ideal parameter for risk stratification in the context of AR.

Despite the short-term reduction of LV volume in the first year after AVR, eccentric remodeling and volume overload in patients with AR causes long-term myocardial damage. Most of the AR patients got surgery in late stage of the disease, reflected by a median LV-EDVI of 139 ml/m^2^ with severely dilated LV. The damage of the myocardium becomes apparent after many years i.e., the early changes of LV geometry are not paralleled by a recovery of the systolic function. Multiple factors contribute to the remodeling in AR and the guidelines recognize the importance to reliably identify myocardial damage in a very early stage of AVD. Classic parameters like LVEF, LV end-diastolic and end-systolic diameter, or patient's symptoms, however, seem to be unable to predict sustained myocardial damage in AR patients.

The impact of fibrosis on remodeling in AR is well-established, yet our findings on LGE underline the importance of a broader spectrum of imaging parameters to detect myocardial damage (39, 40). In our cohort, LV strain values reflected well the functional decline in AR 10 years after AVR. In particular, circumferential strain might indicate irreversible damage in AR and could be addressed in future trial design (41, 42). Intraventricular hemodynamic forces integrate strain and LV volumes and do also provide comprehensive information on LV dysfunction (22, 25). The clinical implications of IVPG over time remains to be defined, since their prognostic implications remain unknown.

### Conclusions

In this study, CMR was providing valuable insights into long-term LV remodeling after AVR and provided information on myocardial changes over a 10-year follow up period. CMR was a suitable method for disease monitoring and guiding regardless of the underlying AV pathology.

Overall, AVR provided improvement of LV function and physical capacity were seen regardless of the underlying AVD at short to intermediate FU. However, patients with AR suffered from long-term alteration of myocardial function compared to AS patients. No long-term improvement in circumferential strain and a trend toward long-term LV dilatation was observed in AR. In AS patients, LV longitudinal strain improved to normal levels and the LV geometry was more robust over the 10-year period. At the same time, AS patients presented significantly more LGE over time than AR patients. Close monitoring of patients with AVD seems indispensable to avoid irreversible structural damage of the heart and to perform AVR at an appropriate stage. Further research is needed to establish imaging-biomarkers and to improve care for patients with AVD.

## Limitations

The sample size of our study is small, particularly regarding the AR group. Further studies are indispensable to confirm our results. Moreover, this study reports only on male patients. Our work is of explorative nature. We aimed to generate hypotheses which ultimately serve for future trial design in the field of valvular heart disease. No sample size calculation has been performed. The temporal development of multiple parameters has been assessed. To control for Type I error, Bonferroni correction of *p*-values has been applied, what in turn increases the risk of Type II errors. Thus, *p*-values should be interpreted with caution. The lack of ECV and T1/T2 mapping is another important limitation.

## Data Availability Statement

The datasets generated during and analyzed during the current study are available from the corresponding author on reasonable request.

## Ethics Statement

The studies involving human participants were reviewed and approved by Charité-Universitätsmedizin Berlin: EA2/077/14. The patients/participants provided their written informed consent to participate in this study.

## Author Contributions

NR, LS, and SK wrote the main manuscript text. All authors reviewed the manuscript.

## Conflict of Interest

SK, AM, VF, and BP received support from the DZHK (German Center for Cardiovascular Research), Partner Site Berlin. SK was supported by Philips Healthcare and received speaker honoraria from Medis. CS is an employee of Philips Healthcare. VF has relavant (institutional) financial activities outside the summited work with following commercial entities: Medtronic GmbH, Biotronik SE & Co., Abbott GmbH & Co. KG, Boston Scientific, Edwards Lifesciences, Berlin Heart, Novartis Pharma GmbH, JOTEC GmbH and Zurich Heart in relation to educational grants (including travel support), fees for lectures and speeches, fees for professional consultation and research & study funds. AM declares the receipt of consulting and lecturing fees from Medtronic GmbH and Edwards Lifesciences Services GmbH, and consulting fees from Pfizer. FS declares the receipt of honoraria, consultancy fees or travel support from Medtronic GmbH, Biotronik SE & Co., Abbott GmbH & Co. KG, Sanofi S.A., Cardiorentis AG, Novartis Pharma GmbH. The remaining authors declare that the research was conducted in the absence of any commercial or financial relationships that could be construed as a potential conflict of interest.

## Abbreviations

AR: Aortic regurgitation
AS: Aortic stenosis
AVD: Aortic valve disease
AVR: Aortic valve replacement
AVS: Acquired voxel size
CMR: Cardiac magnetic resonance imaging
ECV: extracellular volume
EDV: End-diastolic volume
EDVI: End-diastolic volume index
EF: Ejection fraction
ESV: End-systolic volume
ESVI: End-systolic volume index
FU: Follow-up
HemForces: Hemodynamic forces
IVPG: Intraventricular pressure gradients
LGE: Late Gadolinium Enhancement
LV: Left ventricle
LVEF: Left ventricular ejection fraction
LVMI: Mass index
MyoGCS: Global myocardial circumferential strain
MyoGLS: Global myocardial longitudinal strain
RVS: Reconstructed voxel size
SI: Sphericity index
TAVR: Transcatheter aortic valve replacement
TE: Echo time
TR: Time to repetition
4Ch: Four chamber.
