# Supplementary material for: Assessment of 10-Year Left-Ventricular-Remodeling by CMR in Patients Following Aortic Valve Replacement
Source: Front Cardiovasc Med. 2021 Mar 22;8:645693. doi: 10.3389/fcvm.2021.645693 (PMC8019709; doi:10.3389/fcvm.2021.645693)
Supplement: Supplementary file 1 [file Data_Sheet_1.pdf]

Supplementary Material

Manuscript title: Assessment of 10-Year Left-Ventricular- Remodeling by CMR in Patients Following Aortic Valve Replacement

i.) Supplementary Figures

Figure S1

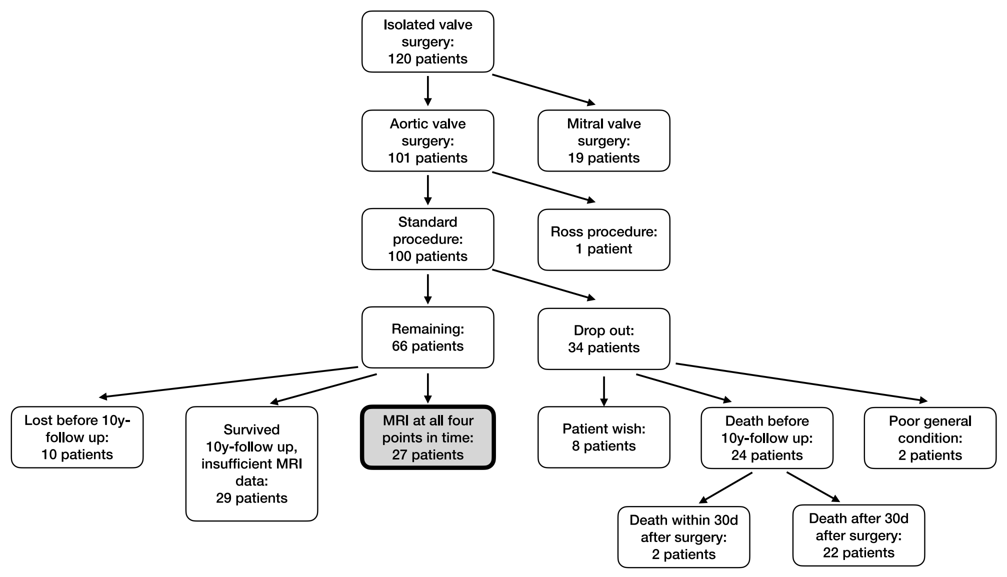

Figure S1. Diagram illustrating the main reasons for drop out in our study cohort.

Figure S2

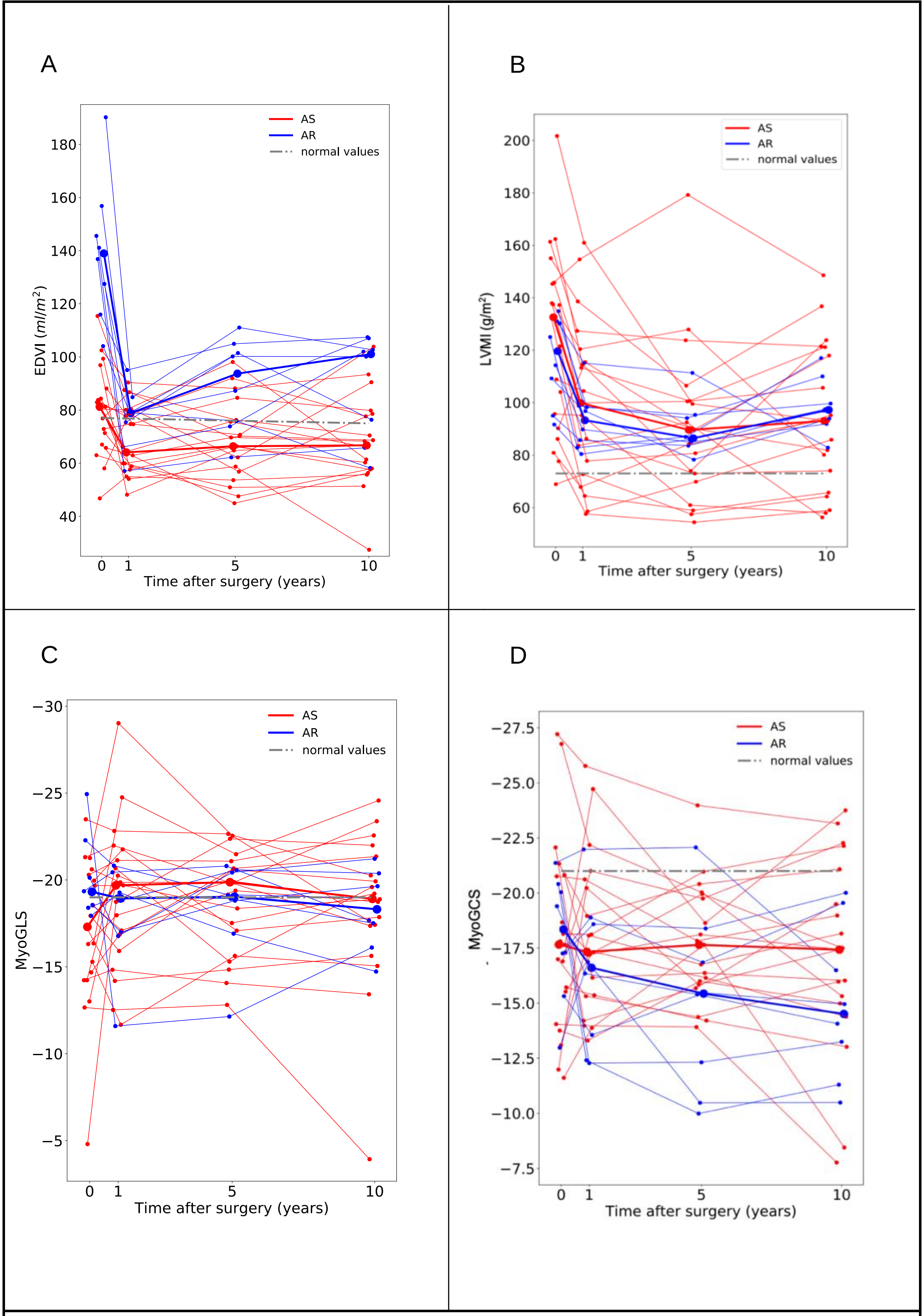

Figure S2. Illustration of the individual responses of the myocardium following aortic valve replacement. Four specific parameters are displayed over time to illustrate response for both AS and AR patients. A: EDVI, end-diastolic volume index; B: LVMI, left ventricular mass index; C: MyoGLS, myocardial global longitudinal strain; D: MyoGCS, myocardial global longitudinal strain. Bold dots represent the median values, respectively.

ii.) Supplementary Tables

Table S1. Comparison between both groups (aortic stenosis and aortic regurgitation) at all four assessed points in time for each variable respectively (unadjusted p-values). EDVI = end-diastolic volume index, ESVI = end-systolic volume index, FU = follow up, HemForces = hemodynamic forces, MyoGCS = myocardial global circumferential strain, MyoGLS = myocardial global longitudinal strain, LVEF = left ventricular ejection fraction, LVMI = left ventricular mass index, SI = sphericity index, SPC = subjective physical capacity, ΔPm = mean pressure gradient across the aortic valve. Unadjusted (top) and adjusted (bottom) p-values are shown.

Variable	FU = 0 years	FU = 1 year	FU = 5 years	FU = 10 years
NYHA	0.953 / 1.000	0.553 / 1.000	1.000 / 1.000	1.000 / 1.000
SPC	0.288 / 1.000	0.484 / 1.000	0.527 / 1.000	0.085 / 1.000
EDVI	< 0.001 / < 0.001	0.180 / 1.000	0.007 / 0.297	0.022 / 0.954
ESVI	< 0.001 / < 0.001	0.051 / 1.000	0.016 / 0.693	0.016 / 0.693
LVMI	0.515 / 1.000	0.658 / 1.000	0.775 / 1.000	0.481 / 1.000
SI	< 0.001 / 0.018	0.238 / 1.000	0.034 / 1.000	0.019 / 0.815
LVEF	0.180 / 1.000	0.058 / 1.000	0.217 / 1.000	0.119 / 1.000
MyoGLS	0.093 / 1.000	0.515 / 1.000	0.658 / 1.000	0.696 / 1.000
MyoGCS	0.856 / 1.000	0.283 / 1.000	0.106 / 1.000	0.180 / 1.000
HemForces	< 0.001 / < 0.001	0.064 / 1.000	0.097 / 1.000	0.033 / 1.000
ΔPm	0.004 / 0.154	0.692 / 1.000	0.927 / 1.000	0.846 / 1.000

Table S2. Comparison between individual points in time for each parameter and patients with aortic stenosis. EDVI = end-diastolic volume index, ESVI = end-systolic volume index, FU = follow up, HemForces = hemodynamic forces, MyoGCS = myocardial global circumferential strain, MyoGLS = myocardial global longitudinal strain, LVEF = left ventricular ejection fraction, LVMI = left ventricular mass index, SI = sphericity index, SPC = subjective physical capacity, ΔPm = mean pressure gradient across the aortic valve. Unadjusted (top) and adjusted (bottom) p-values are shown.

Variable	Group	FU = 0 and 1 year	FU = 0 and 5 years	FU = 0 and 10 years	FU = 1 and 5 years	FU = 5 and 10 years	FU = 1 and 10 years
NYHA	AS	0.005 / 0.317	< 0.001 / 0.007	0.176 / 1.000	0.483 / 1.000	0.028 / 1.000	0.175 / 1.000
SPC	AS	< 0.001 / 0.020	0.016 / 1.000	0.014 / 0.898	0.120 / 1.000	0.765 / 1.000	0.183 / 1.000
EDVI	AS	0.010 / 0.640	0.006 / 0.383	0.007 / 0.469	0.715 / 1.000	0.948 / 1.000	0.811 / 1.000
ESVI	AS	0.018 / 1.000	0.061 / 1.000	0.048 / 1.000	0.159 / 1.000	0.665 / 1.000	0.483 / 1.000
LVMI	AS	< 0.001 / < 0.001	< 0.001 / < 0.001	< 0.001 / < 0.001	0.027 / 1.000	0.437 / 1.000	0.317 / 1.000
SI	AS	0.335 / 1.000	0.724 / 1.000	0.021 / 1.000	0.316 / 1.000	0.026 / 1.000	< 0.001 / 0.007

LVEF	AS	0.125 / 1.000	0.985 / 1.000	0.494 / 1.000	0.086 / 1.000	0.386 / 1.000	0.190 / 1.000
MyoGLS	AS	0.060 / 1.000	0.077 / 1.000	0.153 / 1.000	0.910 / 1.000	0.917 / 1.000	0.930 / 1.000
MyoGCS	AS	0.801 / 1.000	0.949 / 1.000	0.951 / 1.000	0.842 / 1.000	0.792 / 1.000	0.796 / 1.000
HemForces	AS	0.791 / 1.000	0.429 / 1.000	0.741 / 1.000	0.364 / 1.000	0.708 / 1.000	0.449 / 1.000
ΔPm	AS	< 0.001 / < 0.001	< 0.001 / < 0.001	< 0.001 / < 0.001	0.480 / 1.000	0.930 / 1.000	0.460 / 1.000

Table S3. Comparison between indvidual points in time for each parameter and patients with aortic regurgitation. EDVI = end-diastolic volume index, ESVI = end-systolic volume index, FU = follow up, HemForces = hemodynamic forces, MyoGCS = myocardial global circumferential strain, MyoGLS = myocardial global longitudinal strain, LVEF = ejection fraction, LVMI = left ventricular mass index, SI = sphericity index, SPC = subjective physical capacity, ΔPm = mean pressure gradient across the aortic valve. Unadjusted (top) and adjusted (bottom) p-values are shown.

Variable	Group	FU = 0 and 1 year	FU = 0 and 5 years	FU = 0 and 10 years	FU = 1 and 5 years	FU = 5 and 10 years	FU = 1 and 10 years
NYHA	AR	0.262 / 1.000	0.078 / 1.000	0.048 / 1.000	0.463 / 1.000	0.317 / 1.000	0.780 / 1.000
SPC	AR	0.982 / 1.000	0.731 / 1.000	0.617 / 1.000	0.581 / 1.000	< 0.001 / 0.040	0.061 / 1.000
EDVI	AR	< 0.001 / < 0.001	< 0.001 / < 0.001	< 0.001 / < 0.001	0.004 / 0.264	0.760 / 1.000	0.085 / 1.000
ESVI	AR	< 0.001 / < 0.001	0.018 / 1.000	0.001 / 0.092	0.044 / 1.000	0.863 / 1.000	0.159 / 1.000
LVMI	AR	< 0.001 / < 0.001	< 0.001 / < 0.001	0.077 / 1.000	0.339 / 1.000	0.140 / 1.000	0.353 / 1.000
SI	AR	< 0.001 / 0.013	0.015 / 0.964	0.270 / 1.000	0.085 / 1.000	0.046 / 1.000	< 0.001 / 0.059
LVEF	AR	1.000 / 1.000	0.894 / 1.000	0.584 / 1.000	0.627 / 1.000	0.758 / 1.000	0.499 / 1.000
MyoGLS	AR	0.251 / 1.000	0.593 / 1.000	0.172 / 1.000	0.704 / 1.000	0.495 / 1.000	1.000 / 1.000
MyoGCS	AR	0.228 / 1.000	0.076 / 1.000	0.043 / 1.000	0.119 / 1.000	1.000 / 1.000	0.350 / 1.000
HemForces	AR	0.061 / 1.000	0.263 / 1.000	0.053 / 1.000	0.277 / 1.000	0.620 / 1.000	0.463 / 1.000
ΔPm	AR	1.000 / 1.000	1.000 / 1.000	1.000 / 1.000	0.785 / 1.000	0.597 / 1.000	0.801 / 1.000

Table S4. Number of patients with a deterioration of ≥ 2 percentage points in global myocardial circumferential (MyoGCS) and longitudinal strain (MyoGLS) from baseline to 10-year follow up in dependency of the presence of late gadolinium enhancement (LGE) at baseline for patients with aortic stenosis.

Aortic Stenosis		LGE at FU = 0 years	
		yes	no
Deterioration in MyoGCS of ≥ 2 percentage points from baseline to FU = 10 years	yes	1	6
	no	4	8
Deterioration in MyoGLS of ≥ 2 percentage points from baseline to FU = 10 years	yes	2	3
	no	3	11

**Table S5.** Number of patients with a deterioration of ≥ 2 percentage points in global myocardial circumferential (MyoGCS) and longitudinal strain (MyoGLS) from baseline to 10-year follow up in dependency of the presence of late gadolinium enhancement (LGE) at baseline for patients with aortic regurgitation.			
Aortic Regurgitation		LGE at FU = 0 years	
		yes	no
Deterioration in MyoGCS of ≥ 2 percentage points from baseline to FU = 10 years	yes	0	5
	no	0	3
Deterioration in MyoGLS of ≥ 2 percentage points from baseline to FU = 10 years	yes	0	3
	no	0	5

Table S6. Detailed information on the cohort of eight (n = 8) patients with aortic regurgitation (AR) illustrating valve pathology, AR severity, type of prosthetic valve used, and evolution of Prosthetic valve (PrV) over the 10-year follow-up period. AR = Aortic regurgitation; PG = pressure gradient; PrV = Prosthetic valve; Pt. = patient; Re-OP = Reoperation

AR Pt.	Leading Aortic valve pathology	Valve structure	AR severity	Prosthetic valve (PrV)	10-Year PrV Mean PG (mm Hg)	10-Year PrV Max. PG (mm Hg)	10-Year PrV function	10- Year PrV Re-OP
1	Bicuspid valve	calcified	severe	bioprosthetic	21	36	good	no
2	Cusp prolapse	normal	severe	bioprosthetic	24	39	degenerated	yes (10-y FU)
3	Endocarditis	thickened	moderate	mechanical	7	14	good	no
4	Aortic enlargement	calcified	severe	mechanical	9	15	good	no
5	Endocarditis	thickened	severe	mechanical	14	25	good	no
6	Aortic enlargement	calcified	moderate	mechanical	18	26	good	no
7	Bicuspid valve	calcified	severe	mechanical	11	17	good	no
8	Cusp prolapse	normal	severe	mechanical	14	27	good	no

Table S7. Detailed information on the cohort of nineteen (n = 19) patients with aortic stenosis (AS) illustrating valve pathology, AS severity, type of prosthetic valve used, and evolution of aortic valve disease/replacement over a 10-year follow-up period. AS = Aortic stenosis; PG = pressure gradient; PrV = Prosthetic valve; Pt. = patient; Re-OP = Reoperation

AS Pt.	Leading Aortic valve pathology	Valve structure	AS severity	Prosthetic valve (PrV)	10-Year PrV Mean PG (mm Hg)	10-Year PrV Max. PG (mm Hg)	10-Year PrV function	10- Year PrV Re-OP	> 10-Year PrV Re-OP
1	AS	calcified	severe	bioprosthetic	22	33	degenerated	no	no
2	AS	calcified	severe	bioprosthetic	10	26	good	no	no
3	AS	calcified	severe	bioprosthetic	15	24	degenerated	no	n/a
4	AS	thickened	severe	bioprosthetic	27	50	degenerated	no	yes (12-y FU)
5	AS	calcified	moderate	bioprosthetic	12	23	degenerated	yes (8-y FU)	no
6	AS	calcified	severe	bioprosthetic	11	19	good	no	n/a
7	AS	calcified	severe	bioprosthetic	29	40	good	no	n/a
8	AS	calcified	severe	bioprosthetic	45	60	degenerated	no	n/a
9	AS	calcified	severe	bioprosthetic	12	23	degenerated	no	n/a
10	AS	calcified	severe	bioprosthetic	10	15	degenerated	no	yes (12-y FU)
11	AS	calcified	severe	bioprosthetic	6	10	good	no	n/a

12	AS	calcified	severe	bioprosthetic	7	13	degenerated / AR	yes (8-y FU)	no
13	AS	calcified	severe	bioprosthetic	8	13	Endocarditis at 1-y FU	yes (1-y FU)	no
14	AS	calcified	severe	bioprosthetic	18	28	degenerated	no	n/a
15	AS	calcified	severe	bioprosthetic	11	12	degenerated / AR	no	n/a
16	AS	calcified	severe	mechanical	12	17	good	no	n/a
17	AS	calcified	severe	mechanical	15	28	good	no	n/a
18	AS	calcified	severe	mechanical	15	27	good	no	n/a
19	AS	calcified	severe	mechanical	16	26	good	no	n/a
